# In this month’s Bulletin

**DOI:** 10.2471/BLT.25.000625

**Published:** 2025-06-01

In the editorial section, Kinda Alsamara and David Forbe (350) explain the difficulty of translating “well-being” from English to Arabic. Ankita S Achanta and Ther W Aung (351) ask how to manage the loss of publicly available research datasets. 

## Cameroon 

### Healthy neonates

Gaelle Vofo et al. (375–382) screen babies for hearing and sight.

## Democratic Republic of the Congo

### Epidemiology of hepatitis B infections in children

Camille E Morgan et al. (354–365) analyse demographic and health survey data.

## Equatorial Guinea

### Malaria control

Guillermo A García et al. (392–402) test indoor residual spraying coverage targets. 

## Senegal 

### Estimates of female genital mutilation

Kathrin Weny et al. (366–374) reveal implications of different reporting methods.

## Global

### Health laboratory licensing

Markus Huber et al. (383–391) analyse best practices.

### World Health Organization and research ethics

Carl H Coleman et al. (403–409) present a tool for assessing oversight systems.

### Caesarean deliveries when needed

Tanya Doherty et al. (410–412) argue for midwifery models of care.

